# Artificial intelligence for the detection, prediction, and management of atrial fibrillation

**DOI:** 10.1007/s00399-022-00839-x

**Published:** 2022-02-11

**Authors:** Jonas L. Isaksen, Mathias Baumert, Astrid N. L. Hermans, Molly Maleckar, Dominik Linz

**Affiliations:** 1 Department of Biomedical Sciences, University of Copenhagen, Copenhagen, Denmark; 2 School of Electrical and Electronic Engineering, The University of Adelaide, Adelaide, SA Australia; 3 Department of Cardiology, Maastricht University Medical Center and Cardiovascular Research Institute Maastricht, Maastricht, The Netherlands; 4 Department of Computational Physiology, Simula Research Laboratory, Oslo, Norway

**Keywords:** AF, AI, Machine learning, Neural networks, Disease management, Deep learning, VHF, KI, Maschinelles Lernen, Neuronale Netze, Disease Management, Mehrschichtiges Lernen

## Abstract

The present article reviews the state of the art of machine learning algorithms for the detection, prediction, and management of atrial fibrillation (AF), as well as of the development and evaluation of artificial intelligence (AI) in cardiology and beyond. Today, AI detects AF with a high accuracy using 12-lead or single-lead electrocardiograms or photoplethysmography. The prediction of paroxysmal or future AF currently operates at a level of precision that is too low for clinical use. Further studies are needed to determine whether patient selection for interventions may be possible with machine learning.

## Abstract (de)

In diesem Beitrag wird der aktuelle Stand von Algorithmen des maschinellen Lernens zur Erkennung, Vorhersage und Behandlung von Vorhofflimmern zusammengefasst, zudem werden die Entwicklung und Prüfung von künstlicher Intelligenz in der Kardiologie und anderen Bereichen dargelegt. Nach heutigem Stand lässt sich Vorhofflimmern mithilfe künstlicher Intelligenz in 12-Kanal- oder 1‑Kanal-Elektrokardiogrammen bzw. in Photoplethysmogrammen zuverlässig erkennen. Die Vorhersage von paroxysmalem oder neu auftretendem Vorhofflimmern hat die für den klinischen Einsatz erforderliche Genauigkeit noch nicht erreicht. Weitere Studien sind notwendig, um zu untersuchen, ob auf Basis des maschinellen Lernens eine Patientenselektion für Interventionen möglich ist.

## Background

Atrial fibrillation (AF) is the most common sustained arrhythmia and affects more than 43 million people globally [1]. In the European Union, almost 8 million people >65 years of age had AF in 2016, a number that is expected to increase to over 14 million by 2060 due to increased longevity and increasing prevalence of AF risk factors, which leads to increased costs associated with detection, diagnosis, and management of AF. Within the first year of diagnosis, each AF patient in Germany is associated with a cost of over 2200 € [2]. Screening efforts are costly: data from the Gutenberg Health Study estimated 12-lead electrocardiogram (ECG)-based screening in the 65- to 74-year-old general population to cost approximately 30,000 € per gained quality-adjusted life-year [3].

Artificial intelligence (AI) methods—including machine learning and artificial neural networks (deep learning)—can perform some tasks much faster than human experts and at a level comparable to humans or with even greater precision [4–6]. Nevertheless, recent experience suggests that the performance of AI-based systems often fails once they are implemented in a real-life setting, underlining the importance of a careful approach to AI development and validation [7, 8]. Although AI methods have the potential to improve AF detection and even to reduce costs [9], the exact role of AI in clinical AF management remains unclear.

Thus, the aim of this review article was to summarize the state of the art in AI-based detection and management of AF and to review the important steps necessary for development of AI-based systems.

## Introduction to artificial intelligence

**Fig. 1:**
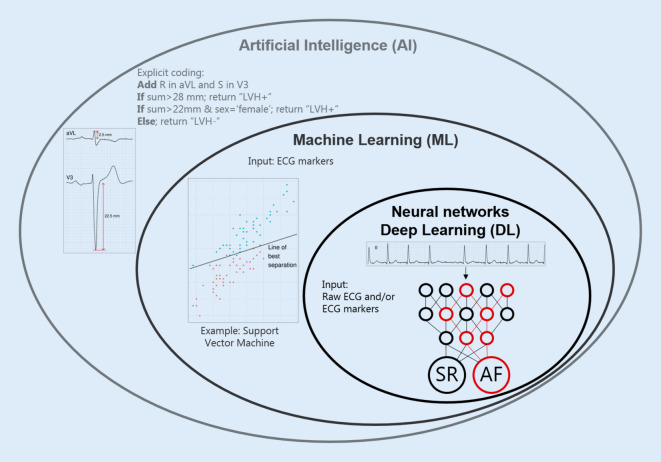
Distinction and overlap between artificial intelligence (*AI*), machine learning (*ML*), and neural networks/deep learning (*DL*). Only DL operates directly on raw waveforms, while other AI methods rely on markers or features. *AI example*: explicit programming to determine whether left ventricular hypertrophy (*LVH*) is present or not based on R and S amplitudes. *ML example*: (imperfect) discrimination between two classes using a support vector machine. *DL example*: Discrimination between sinus rhythm (*SR*) and atrial fibrillation (*AF*) using a simple neural network operating directly on a raw electrocardiogram (*ECG*) rhythm strip

The term “artificial intelligence” was coined at the 1956 summer research workshop at Dartmouth College (Hanover, NH, USA) [10], 6 years after Turing had asked the question “Can machines think?” [11]. The 1956 workshop aimed to develop a machine that could pass the Turing test, i.e., exhibit intelligence at the level of humans. Machines that actually think have not yet been developed, but AI also includes machines that perform simulated thinking, i.e., solve tasks that would otherwise require human intelligence. As such, an automated ECG interpretation software coded line-by-line is within the field of AI (Fig. 1). Machine learning is the subset of AI that relates to self-learning algorithms trained with data, although at times AI is used synonymously with machine learning. Deep learning is a special case of machine learning, whereby information is passed from layer to layer of artificial neurons in a neural network, a process that was inspired by human neuronal computing [12]. The main advantages of artificial neural networks over other machine learning methods are that extraction of specific features is not needed and that the networks are flexible to comprehend complex data. The main limitation of neural networks over other machine learning methods is the need for large, annotated data sets for the model to be successful (i.e., to converge). Many different neural architectures exist; of special interest to AF are recurrent neural networks (RNNs) and convolutional neural networks (CNNs). RNNs are ideal for long time series and forecasting, whereas CNNs are used for image and signal analysis [6, 7, 12]. Many papers referenced in this review used CNNs.

Although AI technically includes programs that are not classified as machine learning, this review will focus on the machine learning part of AI algorithms, and special emphasis is put on neural networks (deep learning).

## Evaluation of the performance of AI algorithms

Metrics for evaluation of machine learning algorithms are the same as for any other algorithm or diagnostic test, however often a condition may be rare, and researchers should be aware of pitfalls among some metrics in this case.

**Fig. 2:**
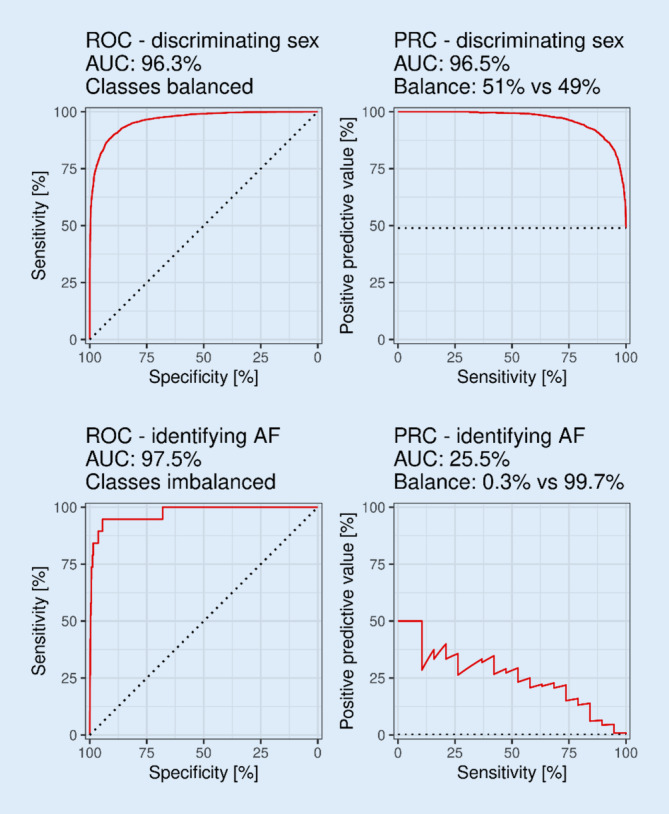
*Top*: Model performance is similarly displayed by the receiver-operator characteristics (*ROC*) curve and the precision-recall curve (*PRC*) when classes are balanced. *Bottom*: Poor model performance combined with imbalanced classes results in a good-looking ROC, but the PRC curve reveals the poor performance. For PRC curves, positive predictive value is often termed precision, and sensitivity termed recall. (*AF* atrial fibrillation, *AUC* area under the curve)

For classification tasks, the standard confusion matrix-derived metrics of sensitivity, specificity, positive predictive value (PPV), and negative predictive value (NPV) are useful. Accuracy (the share of correct predictions out of total predictions) is a good metric only in balanced datasets, but may not reflect the diagnostic performance in imbalanced datasets (i.e., rare events). To illustrate this issue: if an event occurs in one out of 1000 people, a model that predicts the event never to occur would achieve a seemingly good accuracy of 99.9%. In imbalanced datasets, model performance may be suboptimal despite high sensitivity and specificity. One paper reported a sensitivity of 79.0% and a specificity of 79.5% for the prediction of AF, but the PPV (not reported) was 26.1% [13]. For imbalanced datasets, the PPV should be reported along with sensitivity and specificity, and the accuracy metric should be replaced by the F1 score, which is the harmonic mean of sensitivity and PPV and may more accurately reflect the performance of the model [4, 14]. In the aforementioned paper, the reported accuracy and the F1 score were 79.4 and 39.2%, respectively. The area under that receive-operator characteristics (ROC) curve (AUC), also termed the C‑statistics, is based on sensitivity and specificity and is unsuitable for algorithms evaluated on imbalanced datasets [14] (in the previously mentioned paper [13], AUC was 0.87). The ROC curve should be replaced by or supplemented with the precision-recall curve (PRC), whereby the axes are precision (PPV) and recall (sensitivity) (see Fig. 2). The ROC-based AUC/C-statistic should be replaced by the corresponding area under the PRC (sometimes termed AUPRC) [14].

For regression tasks, the prediction error is often quantified using the mean absolute error (MAE) or the root-mean-squared error (RMSE) [6]. The RMSE metric punishes outliers more heavily than the MAE metric, and a large difference between MAE and RMSE thus indicates that extreme outliers exist, which may be caused by catastrophic predictions or large ground truth errors. The prediction error may also be visualized with a “predicted vs. ground truth” scatter plot including the identity line, often accompanied by a correlation coefficient, or a Bland–Altman plot [15].

## AI development

To develop machine learning models, a large dataset is typically required. In supervised machine learning (i.e., with labelled data), a dataset is typically split into two parts: training and testing. This is a crucial step to avoid over-fitting, which is the phenomenon whereby the model is not just trained to use the overall characteristics of a biological signal or measurement, but starts to infer relations to the underlying population. A consequence of over-fitting is that the model will perform poorly in other unseen populations.

Another frequently used strategy to avoid over-fitting is cross-validation, whereby the data is split randomly into a number of subsets, for instance, five equally sized sets. The same underlying model is then trained in five replications, each using a different 20% of the data for testing/validation and the remaining 80% of the data for training. This method uses the entire dataset, is unbiased, and furthermore allows the researcher to assess model stability.

Despite the use of train-test split or cross-validation, over-fitting may occur if the training is done repeatedly and the test error is used to select the optimal hyperparameters (fine tuning for machine learning) or to optimize network architecture (deep learning). In this case, a validation set should be identified to assess the performance of the final model and to ensure the collection of the correct metrics.

Regardless of the training strategy, value is added if machine learning models are validated in an external population to show that they generalize well. Even though this step does not guarantee that AI works as intended in general practice, it arguably improves the likelihood that the developed model will be useful upon implementation [7].

## AF detection with AI

The current guidelines of the European Society of Cardiology on the Diagnosis and Management of Atrial Fibrillation require a 12-lead ECG or >30‑s single-lead ECG documentation for a definitive diagnosis of AF [1]. However, the guidelines also highlight the need for screening, risk tools, and prediction models for AF and specifically mention the use of both mobile health (mHealth) technology and AI.

AF detection using AI works fairly well on 12-lead ECGs, and the main source of false-positive detections is premature atrial contractions and pronounced respiratory arrhythmia, which frequently occurs in young individuals. Using standard 12-lead ECGs, Cai et al. applied neural networks to obtain an F1 score of 95–96% (99% for AF vs sinus rhythm, excluding other rhythms) [16]. Jo et al. developed a neural network with a sensitivity of 98.5% and a PPV of 95.4% [17]. They furthermore showed good generalization to external datasets and achieved sensitivities of 99.6–99.9% and PPVs of 91.4–98.0%. Interestingly, the performance of the model was only moderately reduced using lead I only, suggesting that this model may also be used for wearables. Baalman et al. used extensive pre-processing to single heart cycles and then found that lead II and lead V3 were the best candidates for single-lead AF detection using deep learning [18]. However, this strategy only achieved F1 scores of at most 94%. Despite good performance, a definite diagnosis of AF currently needs to be confirmed by a physician. However, 12-lead AI may serve as an initial filter to reduce the number of false-positives, as was done with success for implantable loop recorders [19].

**Fig. 3:**
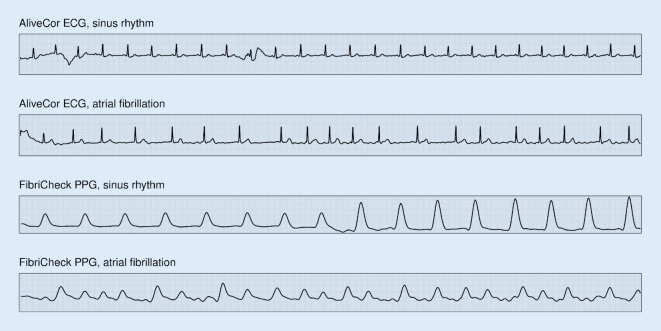
Examples of sinus rhythm and atrial fibrillation recorded using a single lead KardiaMobile (AliveCor) electrocardiogram (*ECG*) device (*top*) and a smartphone-based FibriCheck photoplethysmography (*PPG*, *bottom*). Recordings are from different individuals

Single-lead ECG recordings for AF detection (see Fig. 3) are gaining more interest, since they can be performed by several available mHealth devices and wearables [20]. Hannun et al. obtained 91,232 30‑s, single-lead ECGs from patch devices on >50,000 people and trained a neural network to identify 12 unique rhythms [4]. In a test set of 328 patients, they showed that their network performed better than a group of cardiologists, for multiple arrhythmias including AF. For the 2017 Computing in Cardiology (CinC) Challenge, teams competed to provide the best AF detection among 3658 hidden recordings or varying lengths made with a KardiaMobile single-lead device (a training set with 8528 similar ECGs were available) [21]. There are four classes; sinus rhythm, AF, other rhythm, and noise. Most algorithms generalized with a drop in F1 score (indicating over-fitting to the training set) to a maximum F1 of 83.1%. A voting approach among competing algorithms was better than any individual algorithm and improved F1 to 86.7%. In 2021, a combination of state-of-the-art algorithms improved the F1 score to 90% [22]. The CinC Challenge dataset is freely available and has been frequently used for algorithm development or validation [23–25].

Single-lead ECG may be integrated into a smartwatch wristband, making it widely available, and one study found a high sensitivity (97.7%) albeit a low PPV (40%) validated against an implantable cardiac monitor [26]. Cloud-based AI analysis of wearable ECG may allow continuous updates to the algorithm and facilitate contact with healthcare personnel [27]. Deep learning appears to be a stronger tool than classical machine learning for single-lead ECG analysis including detection of AF [28], and single-lead ECG may play a prominent role in AF detection in the future.

Photoplethysmography (PPG) allows continuous measurement of heart rate using a smartwatch or a smartphone camera through the delineation of the pulse wave (see Fig. 3) [29]. Chen et al. compared wristband PPG and ECG measurements and found slightly higher sensitivity and PPV for the ECG-based measurements [30]. Although their neural network was faster than cardiologists at analyzing wristband PPG or ECG, it was not significantly better. Tison et al. evaluated a smartwatch-based PPG against a 12-lead ECG in 51 patients undergoing cardioversion with a sensitivity of 98% and a PPV of 91% [31]; interestingly, the authors showed that feeding the raw sensor data to a neural network was better than feeding (processed) heart rate data only [32]. Poh et al. similarly evaluated a neural network on 1013 patients with a sensitivity of 95.2% and a PPV of 72.3%, which increased to 100% sensitivity and 87.5% PPV with three measurements from each patient [33]. Taking a clever approach to neural network analysis, whereby they first trained a network to compress the wrist-based PPG signals and then repurposed the network for AF detection, Torres-Soto and Ashley got an F1 score of 96% [34]. Taken together, AI-powered PPG-based detection of AF is possible today with an acceptable false-positive rate and should now be tested in clinical and daily practice [35].

AI was also used for AF detection in other modalities. Two studies have demonstrated that AI may enable AF detection using video [36, 37]. By extracting a facial PPG signal, they achieved sensitivities of 94% and PPVs of 90–98% in a controlled setting. In a small study of 59 patients with paroxysmal AF, Jiang et al. used AI and ballistocardiograms (vibration measurements on the torso) to detect AF with a sensitivity of 96% and a PPV of 94%, although without external validation [38].

Detection of AF will likely improve in the future with the addition of novel algorithms and additional data sets for development—and with clinical validation of algorithms, we may soon see use in clinical practice. The PRICE study plans to enroll 100,000 patients to develop and validate multi-class arrhythmia detection including AF, and the investigators plan a prospective validation of the algorithm [39]. The Scripps Clinic enrolled 25,458 patients with 12-lead ECGs to train a network for multi-class arrhythmia detection [40]. In a large study with an expected 168,000 participants, patients are randomized to access or no access to AI-ECG interpretation [41]. This study will help quantify the added value of AI in clinical practice in a low-risk setting. Three studies focus on AF detection among people with increased stroke risk. One study is enrolling 2450 patients to transfer an existing AI model to work with the S‑Patch Cardio and then uses the device to detect AF in patients with high stroke risk [42]. The HUA-TUO AF Trial will randomize 1740 stroke survivors without documented AF to a handheld single-lead ECG analyzed by an AI algorithm or to usual care [43]. This study will assess the combination of a device and AI interpretation on AF detection and stroke recurrence in a high-risk population. A Leicester, United Kingdom, group is evaluating whether AI in combination with simulation may help discriminate strokes caused by AF, using magnetic resonance imaging during admission for stroke [44]. However, that study may be underpowered since it plans to enroll only 100 patients.

## AF prediction with AI

One step further than AF detection is AF prediction. Based on clinical risk factors alone, machine learning was unable to improve risk prediction beyond the performance of the CHARGE-AF risk score [45]. Also for post-operative AF, machine learning was not better than a logistic regression model when applied to clinical variables [46]. The FIND-AF study set out to detect new-onset AF using AI and electronic health records in a database of 140,000 patients, and perhaps they can show that neural networks can be successfully applied to risk factors [47].

The added value of AI within prediction probably lies in neural network analysis of ECGs, as well as other signals, and the combination with clinical risk factors. Khurshid et al. used AI analysis of ECGs to predict 5‑year risk of AF at a level comparable to the CHARGE-AF risk score, but the combination of the ECG and risk factors identified those at highest risk of AF [48]. Although predictions improved relatively, there is still a long way to go. With a sensitivity of 80%, the PPV in the internal validation set and the two external validation sets was approximately 17, 12, and 2%, respectively. As described previously, another neural network was able to distinguish paroxysmal AF from no AF using sinus rhythm ECGs with a sensitivity of 79% and a PPV of 26% [13]. When the authors applied that same model to patients with embolic stroke of unknown origin, after changing the detection threshold, they got a similar PPV of 23%, but the sensitivity dropped to 63% [49]. The model may thus help predict incident AF, and this is currently being validated prospectively in the BEAGLE study [50].

## AF management with AI

A number of studies have developed machine learning algorithms to assist physicians of different specialties in the management of AF. One group developed an app with a machine learning backend that improved identification of AF in the emergency department and recommended appropriate anticoagulant treatment [51]. However, Levy et al. were unable to train a machine learning algorithm to dose dofetilide as physicians did, perhaps due to the complexity of the clinical situation and perhaps due to large variations between individual physicians [52].

Efforts have been made on risk stratification. Inohara et al. used unsupervised machine learning to cluster patients into groups that proved to have slightly but significantly different risks of major adverse cardiovascular or neurological events. Wanatabe et al. used machine learning on clinical risk factors to predict thromboembolisms slightly better than a logistic regression model [53]. For outcomes of major bleeding and mortality, respectively, the model was not better than logistic regressions. Samaras et al. developed a similar machine learning algorithm for the outcome of mortality, but they did not compare it to logistic regression, nor did they validate the algorithm externally [54]. Loring et al. analyzed machine learning efforts compared to traditional regression for outcomes of death, bleeding, and stroke, and did not find an added value of machine learning [55]. The research so far does not indicate that machine learning can empower clinical variables much beyond classical point scores or logistic regression.

Signal analysis using machine learning may help identify responders to treatment since the complexity of the signals may be better handled by a neural network than human explicit signal processing. So far, in two studies in patients, machine learning did not aid in identifying responders to direct current cardioversion [56] or pulmonary vein isolation [57], respectively; however, both studies had enrolled too few patients for algorithm development. The AI-PAFA Trial will prospectively randomize 340 AF patients to be evaluated for catheter ablation using an AI algorithm or conventional guideline-based rules [58]. A 2021 simulation study indicated that machine learning algorithms may be able to identify pulmonary vein isolation responders [59]; however, further validation studies are needed. A Swedish study is evaluating the use of smartphone-based PPG to detect spontaneous conversion to sinus rhythm for patients about to undergo direct current cardioversion, which is a step towards improved patient selection [60].

## Inherent bias and implications for implementation

The implementation of AI will impact society and care. AI-based tools reflect the data that they were trained on, and a bias in the training data will generally lead to a biased decision [61]. A fault analysis of a decision tool for recurrent crime risk assessment (COMPAS) showed that false-positive predictions of recurrent crimes occurred more often in black people, whereas false-negative predictions occurred more often in white people [62]. In medicine, similar systematic discrimination was seen for a system that assigned special care to certain patients, for which black patients consequently had to be much sicker compared to white patients in order to be referred [63]. In both cases, the AI system was biased since it was trained on biased data, i.e., history showed that black patients actually did have to be sicker to be referred when physicians made the referrals. Thus, bias in training data can translate into a biased AI system. However, AI may also be a powerful tool to identify and mitigate such bias [61].

AI for signal analysis such as AF detection, AF prediction, or AF management is not prone to such systematic bias compared to AI systems operating on patient record data. However, both types of analyses may be prone to data shift. Data shift is the phenomenon whereby the input parameters change, leading to a different relation between input data and outcomes, and with a corresponding change in AI accuracy [64]. One way data may “change” is due to a shift in the underlying patient population. Under the coronavirus disease 2019 pandemic, a change in the relation between fevers and bacterial sepsis due to a change in patient population led to the breakdown of an AI system for sepsis alerting [64]. In AI for signal analysis, this may happen if the device is changed (e.g., a software update) or replaced with another device, or if screening is employed in a different population (e.g., younger individuals than used for training). Thus, continuous quality assessment is an important part of implementation of AI methods. Transfer learning is the fine-tuning of an existing algorithm [65] and may be a way to overcome the data shift issue, just as transfer learning may be used to adapt an algorithm developed in one population to operate optimally in a different population.

## Conclusions

The clinical application of AI has the potential to contribute to AF care in the digital era. Neural networks applied to 12-lead or single-lead ECG or PPG recordings show good performance in detecting AF. Additionally, AI-based prediction of AF shows more potential when applied to biomedical signals than to clinical variables, but these algorithms need to be further developed. AI may even support identification of treatment responders to rhythm control strategies. The real-life performance of the algorithms must be validated in additional populations, and future studies are warranted before they can be broadly implemented in clinical pathways and screening programs. To realize the full potential of AI, the identification of useful actionable data and the integration of AI-derived information into patient management pathways and treatment decision processes represents an important research area to facilitate the clinical implementation in the future and may help to ensure a patient-oriented focus in AF care. Clinical validation, internal and external replication consistency, and generalizability in various healthcare settings regardless of available resources should be included in future research programs.

## Declarations

For this article no studies with human participants or animals were performed by any of the authors. All studies performed were in accordance with the ethical standards indicated in each case.
